# Protective Effect of Glucosinolates Hydrolytic Products in Neurodegenerative Diseases (NDDs)

**DOI:** 10.3390/nu10050580

**Published:** 2018-05-08

**Authors:** Mohammed Sani Jaafaru, Nurul Ashikin Abd Karim, Mohamad Eliaser Enas, Patrick Rollin, Emanuela Mazzon, Ahmad Faizal Abdull Razis

**Affiliations:** 1 Department of Biochemistry, Kaduna State University, Main Campus, PMB 2339 Kaduna, Nigeria; biojafar@gmail.com; 2 UPM-MAKNA Cancer Research Laboratory, Institute of Bioscience, Universiti Putra Malaysia, 43400 UPM Serdang, Selangor, Malaysia; ck_zimase89@yahoo.com (N.A.A.K.); en_mo2008@yahoo.com (M.E.E.); 3 Université d’Orléans et CNRS, ICOA, UMR 7311, BP 6759, F-45067 Orléans, France; Patrick.Rollin@univ-orleans.fr; 4 IRCCS Centro Neurolesi “Bonino-Pulejo”, Via Provinciale Palermo, Contrada Casazza, 98124 Messina, Italy; emazzon.irccs@gmail.com; 5 Laboratory of Molecular Biomedicine, Institute of Bioscience, Universiti Putra Malaysia, 43400 UPM Serdang, Selangor, Malaysia; 6 Laboratory of Food Safety and Food Integrity, Institute of Tropical Agriculture and Food Security, Universiti Putra Malaysia, 43400 UPM Serdang, Selangor, Malaysia; 7 Department of Food Science, Faculty of Food Science and Technology, Universiti Putra Malaysia, 43400 UPM Serdang, Selangor, Malaysia

**Keywords:** crucifer vegetables, glucosinolates, isothiocyanates, neurodegenerative diseases

## Abstract

Crucifer vegetables, *Brassicaceae* and other species of the order Brassicales, e.g., *Moringaceae* that are commonly consumed as spice and food, have been reported to have potential benefits for the treatment and prevention of several health disorders. Though epidemiologically inconclusive, investigations have shown that consumption of those vegetables may result in reducing and preventing the risks associated with neurodegenerative disease development and may also exert other biological protections in humans. The neuroprotective effects of these vegetables have been ascribed to their secondary metabolites, glucosinolates (GLs), and their related hydrolytic products, isothiocyanates (ITCs) that are largely investigated for their various medicinal effects. Extensive pre-clinical studies have revealed more than a few molecular mechanisms of action elucidating multiple biological effects of GLs hydrolytic products. This review summarizes the most significant and up-to-date in vitro and in vivo neuroprotective actions of sulforaphane (SFN), moringin (MG), phenethyl isothiocyanate (PEITC), 6-(methylsulfinyl) hexyl isothiocyanate (6-MSITC) and erucin (ER) in neurodegenerative diseases.

## 1. Introduction

The elderly population is rapidly expanding with the increase in average life expectancy across the globe. The world is now experiencing the “aging era” which comes with social issues like neurodegenerative diseases (NDDs) [1]. These progressive neurological disorders called NDDs are characterized by selective loss of neurons, and they are strongly connected to brain injury from which recovery is highly uncertain and may eventually lead to death [1]. Selective neuronal loss in particular regions of the brain causes different types of dementia-associated disorders which include, but are not limited to Alzheimer’s disease (AD), Parkinson’s disease (PD), Huntington diseases (HD), amyotrophic lateral sclerosis (ALS), spinal cord injury (SCI), and cerebral ischemia/reperfusion (CIR) [2].

Even though the molecular mechanisms governing the pathogenesis of these disorders remain largely under investigation, other anomalies such as protein miscoding, abnormal cleavage and aggregation of certain proteins [3], cytotoxicity, inflammation, conformational changes and apoptosis have been speculated to be the leading causal factors contributing to the neurological disorders mentioned above [2,4].

Basically, rapid progression of NDDs among the elderly population has led to the growing interest in numerous pharmacological approaches aimed at counteracting and arresting neurological malfunction and its devastating effects [4]. Considering the fact that dementia-related disorders are caused by many factors and that there are no effective medications available for their prevention, efficient intervention strategies using naturally occurring bioactive compounds has, therefore, become the best option [5]. Interestingly, some efforts have been made in the identification of potential agents capable of alleviating the pathology of these dementia-related disorders, and some of these potential agents are plants’ secondary metabolites known as phytochemicals [6].

Phytochemicals have long been recognized for exerting different biological activities including but not limited to antioxidant, anti-inflammatory, antibiotic, anticancer, anti-neurodegeneration among others [7]. A group of promising phytochemicals in terms of neuroprotective effect is constituted by glucosinolates (GLs), producing on hydrolysis isothiocyanates (ITCs), which can be regarded as the active form of GLs [8].

## 2. Glucosinolates (GLs): Sources and Enzymatic Activation

Over 130 characterized molecules known as GLs [9] are a peculiar class of thiosaccharidic metabolites displaying a remarkable structural homogeneity, involving a NO-sulfated D-glucosyl thiohydroximate connected with an aglycon whose constitution, depending on plant species, is the sole structural variant [10,11].

**Figure 1:**
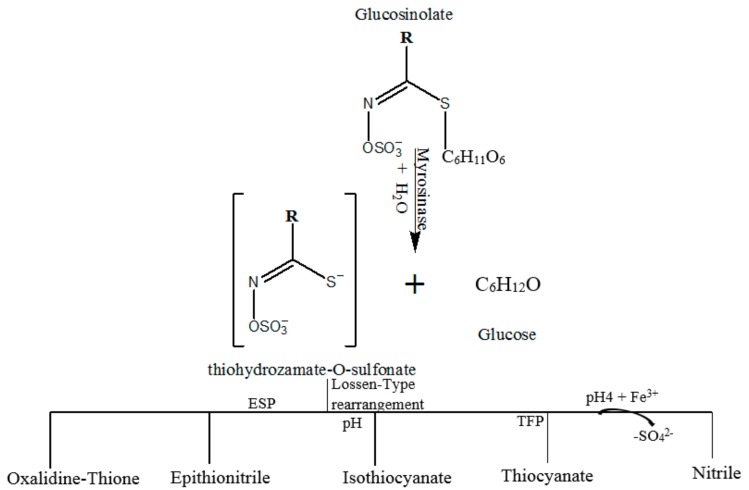
General model of glucosinolates’ hydrolysis by myrosinase to form various specific compounds. ESP and TFP represent epithiospecifier protein and thiocyanate forming protein respectively. The figure was adapted from Fuentes et al. [12].

GLs are predominantly concentrated in seeds, roots and leaves of crucifer vegetables—*Brassicaceae*—and other families of the botanical order Brassicales such as *Moringa oleifera* [12]. In addition to GLs, crucifer vegetables contain atypical glucohydrolase, called myrosinase (EC 3.2.3.147) located in separate cell compartments. When the enzyme and the substrates come in contact in an aqueous environment, myrosinase causes immediate hydrolysis of GLs which, upon glucose release, results in the delivery of unstable aglycon fragments [13]. This, in turn, undergoes structural rearrangements (Figure 1) to form various compounds including ITCs, thiocyanates (TCs), nitriles, epithionitriles and oxazolidine-2-thiones [14,15].

**Figure 2:**
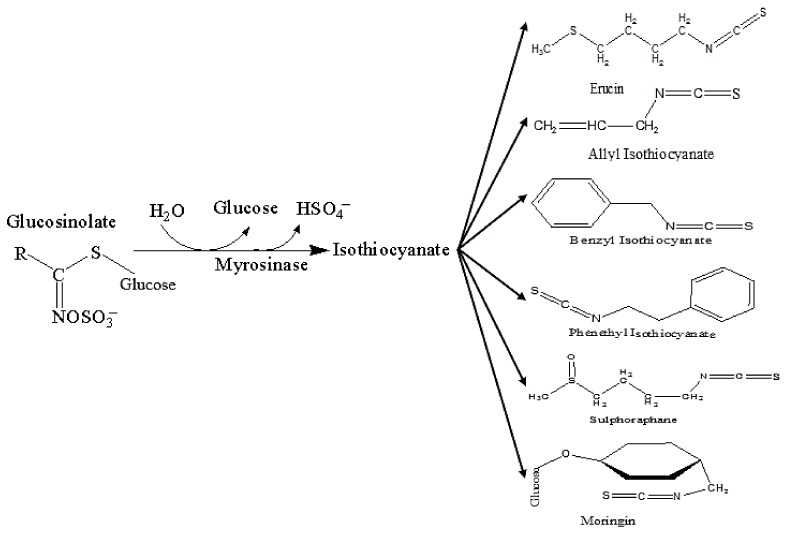
Myrosinase-catalyzed glucosinolate hydrolysis and chemical structure of selected isothiocyanates. Adapted from Sharma and Kapoor [15].

Depending on their aglycon structure, GLs yield various types of ITCs upon myrosinase hydrolysis: glucoraphanin produces sulforaphane (SFN), sinigrin yields allyl isothiocyanate (AITC), gluconasturtiin (GST) gives rise to phenethyl isothiocyanate (PEITC), glucoerucin (GER) results in erucin (ER) production, glucotropaeolin produces benzyl isothiocyanate (BITC) while glucomorigin (GMG) yields moringin (MG) [15,16,17], as shown in Figure 2.

## 3. Isothiocyanates (ITCs) and Their Neuroprotective Effects

As previously mentioned, ITCs are sulfur- and nitrogen-containing phytochemicals obtained from GLs upon myrosinase action. Many studies have revealed the benefits of consuming *Brassicaceae* and *Moringaceae* vegetables, which are the major sources of ITCs. These compounds are believed to contribute to the massive reduction of risk for developing neurodegenerative diseases (NDDs) and some other chronic disorders including but not limited to cardiovascular diseases, various forms of cancer and inflammatory disorders among others [12]. Due to their anti-amyloidogenic, antioxidant, and anti-inflammatory properties, the preventive and treatment ability of ITCs to age and other in vivo and in vitro dementia-related NDDs were extensively explored in the last few years [18,19,20].

### 3.1. Sulforaphane (SFN)

Among the ITCs, SFN, derived from glucoraphanin, has been most widely researched in the recent years [21]. Neuroprotective effects of SFN have been reported in various studies, for its ability to not only arrest many target proteins but also to control various metabolic pathways in neuronal cells [4,21,22]. The beneficial neuroprotective effect of SFN is solely attributed to its ability to activate the nuclear factor erythroid 2–related factor 2/antioxidant response element (Nrf2/ARE) pathway which results in the up-regulation of phase II enzymes [23]. SFN was also reported to decrease the translocation of nuclear factor kappa-light-chain-enhancer of activated B cells (NF-κB) from cytosol to nucleus with a consequent decline in the production of major pro-inflammatory mediators, cytokines, and oxidative markers which stimulate the neuronal apoptotic pathway [24,25,26].

Protective effect and molecular mechanism of action of SFN were investigated in amyloid β (Aβ)-induced oxidative damage and apoptosis in human neuroblastoma cell line (SH-SY5Y) by Lee et al. [27]. SFN pre-treated and Aβ-exposed (after 26 h) cell lines showed evidence of abolishing the occurrence of programmed cell death due to oxidative stress caused by cytotoxic Aβ-fragments [27]. The authors further revealed that, SFN interfered and modulated the ratio of Bcl-2-associated X protein (Bax) to B-cell lymphoma 2 (Bcl-2) proteins and also reduced the level of reactive oxygen species (ROS) by increasing the synthesis of certain enzymes with strong antioxidant activities, such as quinone reductase (NQO1), haem oxygenase (HO-1) and glutamate-cysteine ligase catalytic subunit (GCLC) through initiation of nuclear factor erythroid 2–related factor 2/antioxidant response element (Nrf2/ARE) pathway. In this case, the protective effect of SFN was, therefore, ascribed to the activation of Nrf2/ARE pathway.

Other studies conducted on relevant neuronal cell lines have further demonstrated the protective effect of SFN against Aβ-fragment cytotoxicity through regulation of proteasome system [28,29]. Likewise, Park and Coll reported the protective effect of SFN against Aβ-induced oxidative damage and the increased clearance of the fragments in Neuro-2A and N1E-155 murine neuroblastoma cell lines by increasing the activity of proteasome. The authors also noticed that SFN’s protective activity was stopped when proteasome inhibitors were introduced into the experiment, hypothesizing that SFN elicited its protective activity through the enhancement of Nrf2/ARE pathway, thereby increasing the synthesis of catalytic multi-subunits of proteasome.

A study conducted by Kwak et al. [30] also reported the neuroprotective effect of SFN against hydrogen peroxide (H_2_O_2_) that induced oxidative damage by enhancing the expression of proteasome catalytic subunit 26S. Those similar findings were reflected on another study conducted on HeLa and COS-1 cell lines by Gan et al. [28], who suggested that the up-regulation of proteasome facilitates the clearance of Aβ-aggregates, which in a way resulted in the protein folding improvement in Alzheimer’s disease (AD) model.

Administration of SFN in various doses to AD animal model ameliorated cognitive dysfunction induced by Aβ-fragments, as reported by Kim et al. [31]. Despite the fact that the authors observed less ability of the compound to abolish aggregation of Aβ, the overall effect of SFN in the experiment indicates a modulation of cognitive function through preventive measures on amyloidogenic damage in the brain of the disease models. One year later, a study on animal models of NDDs also disclosed promising results on the therapeutic potential of SFN. For example, oral administration of SFN to AD-like mouse model decreased cholinergic neuronal loss, thereby, ameliorating the cognitive impairment caused by the prior administration of aluminum and D-galactose [32], and the occurrence of these events was ascribed to the ability of SFN to activate the Nrf2 pathway. In a subsequent study, the same authors [33] reported that administration of SFN in the experimental animals significantly decreased the level of Aβ-plaque in both the hippocampal and cerebral cortex of mice, suggesting that SFN could ameliorate damages caused by Aβ-fragment cytotoxicity in AD-mouse model.

Moreover, a study conducted by Lee et al. [34] using scopolamine-induced memory impairment in a mouse model, revealed an interesting neuroprotective effect of SFN against cholinergic deficit and cognitive impairment in the disease model. It was shown that SFN enhances the cholinergic system activity by up-regulating acetylcholine (Ach) and choline acetyltransferase levels in hippocampal and cerebral cortex regions, as well as decreasing acetyl cholinesterase (AchE) activities, which in a way, prevents the setup and escalation of neurodegeneration.

Recently, the role of SFN in okadaic acid (OKA) treated on rats was evaluated by Dwivedi et al. [35] who reported the ameliorative effect of SFN on cognitive impairment. The activity of SFN occurred in this case through decreasing the release of pro-oxidant species: reactive oxygen species (ROS) and reactive nitrogen species (RNS), pro-inflammatory mediators & cytokines: nuclear factor kappa-light-chain-enhancer of activated B cells (NF-κB) and tumor necrosis factor alpha (TNF-α) and inhibiting neuronal programmed cell death in hippocampal and cortex area of the animal brains. The authors further reported the observed up-regulation of Nrf2 and some antioxidant enzymes (GCLC and HO-1) in the tissue samples. When an inhibition study was carried out using Nrf2 small interfering RNA (siRNA) and HO-1 inhibitor, the results indicated the involvement of SFN in the activation of Nrf2 pathway to convey its peculiar neuroprotective activity.

**Figure 3:**
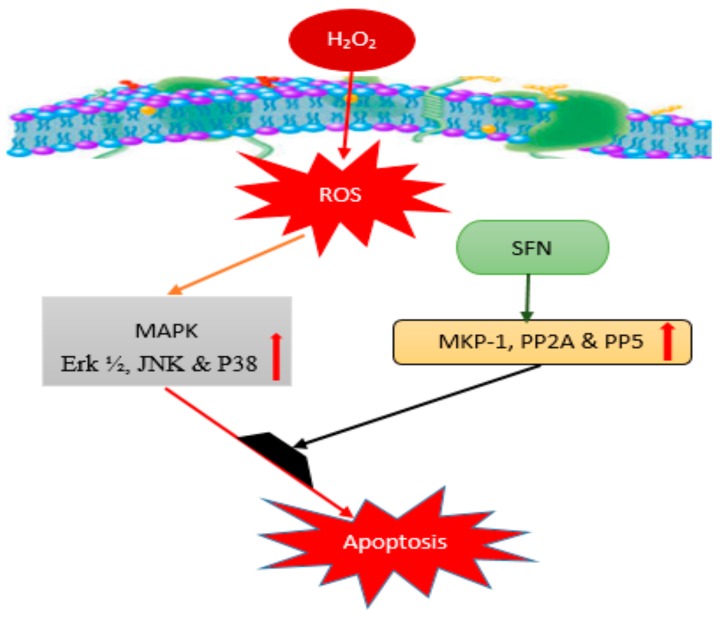
MAPK pathway for neuroprotective effect of sulforaphane: Involving extracellular signal regulated protein kinase ½ (Erk ½), C-Jun N-terminal kinase (JNK), P38, MAPK phosphatase (MKP-1), serine/threonine protein phosphatase 2A (PP2A), Protein Phosphatase 5 (PP5). SFN: sulforaphane; ROS: reactive oxygen species.

SFN was also reported to hamper the progression of Parkinson’s disease (PD) through the interception of pro-inflammatory and apoptotic pathways in a PD animal model induced by intrastriatal injection of 6-hydroxydopamine (6-OHDA) for 28 days [36]. In this light, SFN exhibited its neuroprotective activity against the neurotoxic effect of 6-OHDA on dopaminergic neurons due to its modulatory activation of ERK1/2. Where, the up-regulated phosphatases including mitogen-activated protein kinase phosphatase-1 (MKP-1), protein phosphatase type 2A (PP2A) and protein phosphatase 5 (PP5) suppress the effect of cytokines known as mitogen-activated protein kinase (MAPK) and derivatives, which in turn protect neurons from oxidative damage as demonstrated in Figure 3.

The neuroprotective action of SFN was also demonstrated in acute (2 injections of 40 mg/kg 1-methyl-4-phenyl-1,2,3,6-tetrahydropyridine, MPTP) and sub-acute (5 injections of 20 mg/kg MPTP) models of PD. In particular, the treatment with bioactivated glucoraphanin preserved motor behavior in both acute and subacute MPTP treated mice, prevented dopamine transporter loss and increased tyrosine hydroxylase (TH) expression. These results may be due to at least in part to the release of neurotrophic factors, such as growth associated protein 43 (GAP-43), nerve growth factor (NGF) and brain-derived neurotrophic factor (BDNF), in animals treated with bioactivated glucoraphanin. Moreover, bioactivated glucoraphanin exerted also anti-inflammatory and anti-oxidative actions in both experimental groups, decreasing IL-1β and nitrotyrosine levels while increasing those of Nrf2. All those effects resulted in protection of neurons against apoptosis and consequently in the prevention of dendrite spine loss [37]. In addition, other in vitro studies using different cell lines emphasized various influences of SFN. For instance, Vauzour et al. [38] reported how SFN triggered the mechanism involving nuclear translocation of Nrf2 protein that up-regulates and increases the activity of antioxidant enzymes in *Mus musculus* neuroblastoma (N1E-115) cell line. A similar result was equally obtained by using *Rattus norvegicus* adrenal gland pheochromocytoma (PC-12) as a PD model to assess the neuroprotective effect of SFN [39]. This potent ITC was also reported to abolish events of programmed cell death induced by H_2_O_2_ in a dopaminergic like human neuroblastoma (SH-SY5Y) cell line [40].

As common in other age- and dementia-related NDDs models, the generally reported molecular mechanisms of action of SFN in the in vitro and in vivo PD models is the modulation of Nrf2/ARE pathway, inhibition of pro-inflammatory cascades and abolishment of the apoptotic pathway [36,39,41] as shown in Table 1. However, SFN was reported to prevent blood brain barrier (BBB) dysfunction, preserving tight junction alterations through anti-inflammatory and anti-apoptotic activities [25]. It follows that the preservation of BBB integrity may be another mechanism of action through which SFN exerts its neuroprotective effect. Despite the growing number of in vitro and in vivo studies highlighting neuroprotective properties of SFN, clinical trials to ascertain the efficacy of this ITC in the actual disease condition remain unavailable.

### 3.2. Moringin (MG)

Moringin (MG), i.e., 4-(α-L-rhamnosyloxy) benzyl isothiocyanate, is an ITC of a different source, resulting from myrosinase-catalyzed hydrolysis of glucomoringin (GMG). Due to its potential effect for addressing several disease conditions, MG attracted the attention of a number of researchers in many areas of study including cancer, antimicrobial, NDDs, and so on [16,42,43,44,45]. It was reported that *Moringa oleifera* Lam. leaf extract with a reasonable MG content ameliorates anomalies on memory impairment when investigated in a colchicine-induced AD rat model. The extract provided enough antioxidant activities to overpower oxidative stress conditions set on by colchicine infusion to the animals [46].

Similarly, a study involving an animal model of age-related dementia induced by ethylcholine aziridinium (AF64A) revealed crucial information on the capacity of *M. oleifera* extracts in addressing such disease conditions. Administration of leaf extract in different doses for the period of the experiment improved spatial memory and reduced neuronal degeneration to the minimum level in the hippocampal region of the animal disease model, suggesting that an effect was exerted through the decrease in oxidative damage caused by AF64A and enhancement of cholinergic function [47]. Similarly, *M. oleifera* leaf extract was also reported to cause up-regulation of Cu/Zn superoxide dismutase (SOD), catalase (CAT) and other enzymes with antioxidant properties, thereby boosting their enzymatic activities, which in turn decreased the activities of pro-oxidant enzymes including but not limited to lipid peroxidase as observed in the cerebral cortex and hippocampal regions of AD rat model [48].

Galuppo et al. [44] recently reported the neuroprotective effect of MG on cerebral ischemia/reperfusion (CIR) rat models, with findings clearly indicating that the ITC has the ability to intercept the CIR-induced damage by lowering the release of pro-inflammatory biomarkers including tumor necrosis factor-alpha (TNF-α), nuclear factor of kappa light polypeptide gene enhancer in B-cells inhibitor, alpha (IκB-α) and nuclear factor kappa-light-chain-enhancer of activated B cells (NF-κB). Moreover, MG also revealed its capacity to modulate other oxidative mediators governing the progression of the disease conditions in the experimental model [44]. Another study of the same compound in amyotrophic lateral sclerosis (ALS) transgenic model revealed an obvious interference of MG with the pathophysiology mechanisms that induced the development of the disease. Indeed, MG administration was able to delay the disease onset of about two weeks, probably due to its immunomodulatory, anti-inflammatory, anti-oxidant and anti-apoptotic actions [45]. The data obtained from the MG-treated and -untreated ALS rats displayed a statistically significant difference, therefore, triggering the authors to make an optimistic suggestion on further clinical investigations of the biological effect of MG on ALS human patients.

More so, when this promising ITC was tested on the experimental autoimmune encephalomyelitis mouse model mimicking multiple sclerosis (MS) disease condition, the results clearly indicated the effectiveness of the compound to abolish a series of inflammatory reactions leading to severe pathological MS condition [49]. MG reduced demyelination and axonal loss, it was also found to lower the production of pro-inflammatory markers, such as TNF-α, and oxidative species generation, including nitrotyrosine and inducible nitric oxide synthase (iNOS), that reduced neuronal cell death by modulating the apoptotic pathway through regulating Bax/Bcl-2 ratio. Additionally, MG positively regulated the aberrant Wnt-β-catenin pathway resulting in glycogen synthase kinase 3 beta (GSK3β) inhibition and β-catenin up-regulation, the regulated T cell activation, abolished inflammatory mediators through the activation of peroxisome proliferator-activated receptor gamma (PPARγ). Moreover, MG was able to increase Nrf2 showing an anti-oxidant action [50]. The same authors showed the efficacy of a topical MG cream in reducing inflammation, clinical and histological disease scores, while improving remyelinization in a MS model. Besides, this MG cream was also able to modulate voltage-gated ion channels relieving neuropathic pain and inducing the recovery of hind limbs in a mouse model of MS [51]. Consequently, the authors considered MG as a promising therapeutic agent and potential candidate for the prevention and the treatment of MS.

With a view to further investigate the ability of MG in handling many forms of NDDs, Giacoppo et al. [52] tested the compound for the treatment of spinal cord injury (SCI) in an experimental animal model of the disease condition, where it was found to be able to reduce the severity of histological damage and preserved the normal distribution of reticular fibers in the spinal cord. Moreover, MG triggered an anti-inflammatory process that leads to diminished activation of NF-κB p65 and a reduction of both iNOS expression and apoptosis. MG was, therefore, agreed to exert its effect mainly on the secondary damage to the spinal cord via antioxidant mechanisms of neuroprotection [52].

Likewise, the effects of MG and its non-activated precursor GMG were compared in a mouse model of subacute PD induced by intraperitoneal administration of 1-methyl-4-phenyl-1,2,3,6-tetrahydropyridine (MPTP). The outcome indicated a higher activity of MG compared to that of its precursor in the in vivo aspects of the study, such as body weight changes and in improving motor deficits [50]. Apart from the anti-inflammatory and anti-oxidative stress effects observed in vitro in Murine macrophage cells (RAW 264.7) treated with LPS earlier, the ITC was able to counteract the inflammatory pathway and modulate oxidative stress markers in the animal model. MG also prevented the decrease in TH expression and inhibited apoptosis in vivo [50]. The outstanding effect of MG exhibited in the study encouraged the authors to recommend further harnessing of its potentialities for treatment or prevention of PD in a clinical study.

The effects of MG were also ascertained through various in vitro and in vivo studies; however, there are other aspects of NDDs that need to be explored using this ITC. No clinical investigation involving human subjects to ascertain the efficacy of MG on individuals suffering from the NDDs mentioned above has been reported so far and therefore remains unexplored.

**Figure 4:**
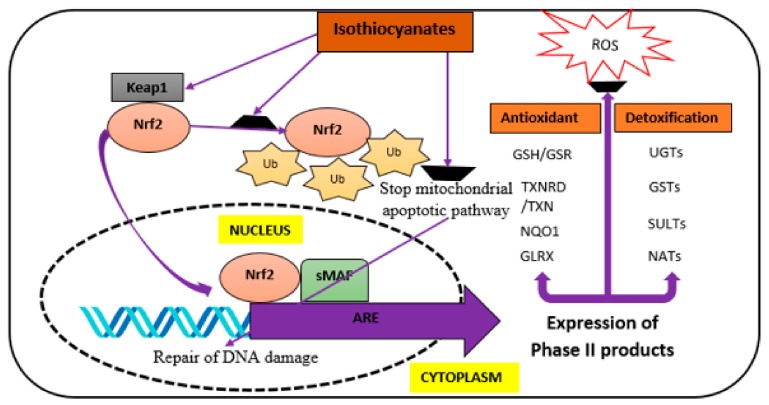
Proposed molecular mechanism of actions through which ITCs elicit their neuroprotective effect. ARE, Antioxidant response element; Glrx, Glutaredoxin; GSH, Glutathione; GSR, Glutathione-disulfide reductase; GSTs, Glutathione *S*-transferase; Keap1, Kelch-like ECH-associated protein 1; NATs, *N*-acetyltransferases; Nrf2, nuclear factor erythroid 2-factor 2; NQO1, NAD(P)H quinone dehydrogenase 1; ROS, Reactive oxygen species; sMAF, proto-oncogene response element; SULTs, Sulfotransferases; TXN, Thioredoxin; TXNRD, Thioredoxin reductase; Ub, Ubiquitin; UGTs, UDP glucuronosyltransferases. Adapted from Giacoppo et al. [19].

In addition, other ITCs have shown certain levels of neuroprotective potential in a growing number of studies [53,54,55]. The less established ITCs also derive from glucosinolates present in the family *Brassicaceae*; gluconasturtiin, glucohesperin and glucoerucin have been characterized and well-studied over the years [13]. The common molecular mechanisms through which ITCs exert their actions include the modulation of Nrf2/ARE, pro-inflammatory and cytokines production, as well as an apoptotic pathway as demonstrated in Figure 4 below.

### 3.3. Phenethyl ITC (PEITC)

PEITC is found predominantly in cruciferous plants, such as watercress or horseradish [56] which contain its precursor 2-phenylethyl GL (gluconasturtiin) [57]. This ITC has shown promising relieving effects in several studies involving oxidative stress and inflammation [53,58,59,60]. In a recent study, Qin et al. [53] claimed that PEITC seems to form conjugates with glutathione (GSH) resulting from cytosolic GSH transferase’s action, thereby modulating the inflammatory effect of iNOS, COX-2 and IL-1β respectively via significant reduction in their expression, in which when not arrested at early stage may result in degeneration of neurons in key areas of brain. PEITC was also reported to influence the expression and nuclear translocation of Nrf2, thus regulating the Nrf2/ARE pathway, and enhancing the expression of phase II enzymes [51] that could vehemently stop the oxidative stress-drive dementia. A study conducted by Boyanapalli et al. [60] using transgenic mice having their Nrf2 gene knocked out and treated with PEITC, demonstrated that the introduction of Nrf2 in the presence of PEITC was crucial to alleviating the severe neuropathological condition in animals.

### 3.4. 6-(Methylsulfinyl) hexyl ITC (6-MSITC)

Besides other long chain molecular congeners, this ITC is produced in wasabi and rocket salad (*Brassicaceae*) through enzymatic hydrolysis of the 6-methylsulfinylhexyl GL precursor (glucohesperin) [61]. 6-MSITC has exhibited impressive properties against many inflammatory diseases and counteractive effects through studies on oxidative stress [53,62,63], which is the major cause of pathology in many neurodegenerative disease conditions. Japanese research groups demonstrated that 6-MSITC inhibits the activation of iNOS and COX-2 by LPS in vitro [62,64]. Similarly, Chen et al. [65] reported the ability of 6-MSITC to regulate the expression of many genes responsible for triggering inflammation and synthesis of certain cytokines via modulating the activity of Nrf2 within the nucleus. The neuroprotective effect of this phytochemical was also tested in vivo, where the up-regulation of phase II enzymes in the liver, known for its detoxification activity, was observed after oral administration of this ITC to the animals. The effect of 6-MSITC was found to be even more pronounced than the previously recorded effects of SFN and MG [66]. The authors further stressed the influence of 6-MSITC for activating Nrf2/ARE detoxification pathway by triggering nuclear translocation of Nrf2 to form a complex with ARE thereby counteracting pro-inflammatory markers’ expression. Even though testing this compound in an animal model of PD revealed astonishing result, Morroni et al. [54] recorded a significant decline in oxidative stress and neuronal death via apoptosis in NDD animal model, thus increasing cognitive function and improving animal behavior. Despite these few recorded studies, the in vivo neuroprotective reports on 6-MSITC are still scarce. Therefore, further exploration of the neuroprotective activity of this ITC, using suitable animals model is crucial.

### 3.5. Erucin (ER)

This ITC is released through enzymatic hydrolysis of its 4-methylsulfanylbutyl GL precursor (glucoerucin), normally found in broccoli sprouts, which is rich GL source. ER can also be formed by in vivo reduction of SFN [67,68]. Even though this compound was poorly investigated for its neuroprotective ability so far, various in vitro studies have reported on its anti-inflammatory and anti-oxidant activities [69,70] which are strongly believed to be connected with neurodegeneration particularly oxidative stress derive NDDs. Yehuda et al. [71] suggested that ER possessed a strong ability to down-regulate the expression of many pro-inflammatory molecules such as TNF-α, IL-1β, and IL-12 in THP-1 cell line. Similarly, Cho et al. [55] reported an inhibitory effect against the NF-κB signaling pathway for ER as an anti-inflammatory agent. In addition to that, in vitro and in vivo studies further revealed the neuroprotective effect of ER, whereby the compound was reported to influence nuclear translocation of Nrf2 and modulate its action through JNK, Erk1/2 and P38 signaling pathway [72], as demonstrated in Figure 3 above. The potential effect of this ITC was also tested against oxidative damage induced by 6-OHDA in neuroblastoma (SHSY5Y) cell, where ER was able to slow down apoptosis by increasing the expression level of GSH, and its anti-oxidant activity [70]. Meanwhile, the respective anti-oxidant, anti-inflammatory and neuroprotective effects exhibited by ER were similar to the other commonly studied ITCs such as SFN and MG. Despite the neuroprotective potential of this ITC, there were very few studies that reported on its promising effects in vivo; as such, there is a high need to further harness those potentials using suitable animal models of NDDs. Table 1 summarized mechanisms of action of SFN, MG, PEITC, 6-MSITC and ER on inflammatory and NDDs including AD, PD, CIR, ALS, MS and SCI employing in vitro and in vivo models.

## 4. Conclusions

Beneficial bio-activities of GLs have drawn the attention of numerous researchers in various areas of life sciences, encompassing both metabolic and non-metabolic disease intervention. In particular, an extensive number of scientific reports have demonstrated the chemopreventive effects of those phytochemicals and their hydrolytic products. Such effects could either be exerted via enhancement of certain processes and pathways directly involved in the disease pathology such as apoptosis in cancerous cells, by direct cell cycle progression arrest, or by initiating a process that leads to complete inhibition of certain metabolic pathways in the cell. In addition, the hydrolytic products of GLs, i.e., ITCs principally, were substantially studied for anticancer activities both at clinical and preclinical levels.

Interestingly, these ITCs have also revealed promising outcomes when tested against several in vitro and in vivo models of NDDs. However, despite all the reports mentioned concerning these chemotherapeutic agents, there remains a need to further explore their preventive or curing potentials for other deadly and incurable ailments, and to further our understanding of the mechanisms and pathways through which each ITC delivers its neuroprotective effect, especially in a well-suited suitable animal model. Additionally, for those compounds, the actual safe and most effective dose required to elicit the desired metabolic response in a suitable animal model, transposable to human subjects, need to be further investigated.

## Figures and Tables

**Table 1:** Summary of the effects and mechanisms of action of sulforaphane (SFN), moringin (MG), phenethyl isothiocyanate (PEITC), 6-(methylsulfinyl) hexyl ITC (6-MSITC) and erucin (ER) on severe inflammatory and neurodegenerative diseases (NDDs).

ITCs or Extract	NDD or Models	Effect on NDDs	Mechanism of Action	Reference
SFN	AD (in vitro) SH-SY5Y Human neuroblastoma cell line	Abolished apoptosis	Modulation of Bax/Bcl2 and Nrf2 pathways	[27]
	AD (in vitro) Neuro-2A & N1E-115 murine neuroblastoma cell line	Increased proteasome activity	Enhancement of Nrf2 pathway	[29]
	AD (in vitro) cell line	Increased proteasome activity	Enhancement of Nrf2 pathway	[30]
	AD (in vitro) HeLa & COS-1 Cell line	Increased proteasome activity & proper folding	Triggering Aβ-fragment’s clearance	[28]
	AD (in vivo) mice induced by AlCl_3_ & D-Galactose	Ameliorated cognitive impairment	Modulation of Nrf2/ARE pathway	[32]
	AD (in vivo) rat model	Improved cognitive function	Modulation of Ach transferase activity	[34]
	AD (in vivo) rat model	Ameliorated cognitive impairment	Modulation of pro-inflammatory production via Nrf2/ARE pathway	[35]
	PD (in vitro) N1E-115 murine neuroblastoma cell line	Abolished apoptotic pathway & improve cognitive function	Modulation of phase II antioxidant enzymes	[38]
	PD (in vitro) PC-12 cell line	Stopped apoptosis	Modulation of pro-inflammatory markers production pathway via Nrf2/ARE pathway	[39]
	PD (in vitro) SH-SY5Y Human neuroblastoma cell line	Abolished apoptosis	Modulation of Nrf2/ARE pathway	[40]
	PD (in vivo) rat model	Decreased the disease progression	Modulation of pro-inflammatory & apoptotic pathway via activation of ERK1/2	[36]
*M. oleifera* crude extract	AD (in vivo) rats colchicine induction	Ameliorated memory impairment	Up-regulation of phase II antioxidant enzymes	[46]
	AD (in vivo) rats Ethyl choline induction	Improved spatial memory and reduce neuronal cell death	Up-regulation of SOD & CAT	[47]
MG	CIR (in vivo) rats model	Improved cognitive function	Modulation of pro-inflammatory biomarkers production & Nfr2/ARE pathway	[44]
	ALS (in vivo) rats model	Delayed the disease onset	Modulation of expression of vital proteins involved in the disease pathology such as Nrf2, iNOS & PARP, and modulation of apoptotic pathway	[45]
	MS (in vivo) mouse model	Abolished series of inflammation	Down regulation of pro-inflammatory & production of oxidative species as well as modulation of apoptotic pathway	[49]
	SCI (in vivo) rats model	Protected neuronal death	Modulation of up-regulated inflammatory markers	[52]
PEITC	NDD (in vitro) cell lines	Abolished inflammation	Initiation of Nrf2 translocation and modulation of Nrf2/ARE signaling pathway	[53]
	NDD (in vivo) transgenic mice model	Alleviated severe pathological condition	Restoration of Nrf2 expression	[60]
6-MSITC	NDD (in vitro) cell lines	Slow down inflammation	Enhancement of Nrf2 activity and slow down expression of pro-inflammatory biomarkers	[65]
	NDD (in vivo) rat model	Stopped inflammation	Enhancement of Nrf2/ARE complex formation and their signaling pathway	[66]
	PD (in vivo) animal model	Decreased apoptosis, increased cognitive function, improved behavior	Modulation of Nrf2/ARE pathway	[54]
ER	NDD (in vitro) cell lines	Stopped inflammation	Counteraction of pro-inflammatory markers’ expression	[71]
	NDD (in vitro) cell lines	Decreased inflammation	Inhibition of NF-κB signaling pathway	[53]
	NDD (in vitro) SH-SY5Y cell lines	Slow down apoptosis	Increase expression of GSH and its activities	[70]
	NDD (in vitro & in vivo) cell lines and animal models	Reduced inflammation	Counteraction of JNK, Erk1/2 and P38 signaling pathway by Nrf2	[72]

NDD represents neurodegenerative diseases; AD, Alzheimer’s disease; PD, Parkinson’s disease; ALS, Amyotrophic lateral sclerosis; MS, Multiple sclerosis; SCI, Spinal cord injury; CIR, Cerebral ischemia/reperfusion; ITCs, Isothiocyanates; SOD, Superoxide dismutase; CAT, catalase; iNOS, inducible nitric oxide synthase; PARP, poly ADP ribose polymerase. NB: severe inflammation lead to up-regulation of cytokines and other pro-inflammatory markers which result in neurodegenerative diseases if not arrested at early stage.
